# Ethnic minorities and COVID-19: examining whether excess risk is mediated through deprivation

**DOI:** 10.1093/eurpub/ckab041

**Published:** 2021-04-07

**Authors:** Cameron Razieh, Francesco Zaccardi, Nazrul Islam, Clare L Gillies, Yogini V. Chudasama, Alex Rowlands, David E Kloecker, Melanie J Davies, Kamlesh Khunti, Thomas Yates

**Affiliations:** 1 Diabetes Research Centre, University of Leicester, Leicester General Hospital, Leicester LE5 4PW, UK; 2 National Institute for Health Research (NIHR) Leicester Biomedical Research Centre (BRC), Leicester General Hospital, Leicester LE5 4PW, UK; 3 Leicester Real World Evidence Unit, Diabetes Research Centre, University of Leicester, Leicester, UK; 4 Clinical Trial Service Unit and Epidemiological Studies Unit (CTSU), Nuffield Department of Population Health, University of Oxford, Oxford, UK; 5 Medical Research Council Epidemiology Unit, University of Cambridge, Cambridge, UK; 6 NIHR Applied Research Collaboration—East Midlands (ARC-EM), Leicester General Hospital, Leicester, UK

## Abstract

**Background:**

People from South Asian and black minority ethnic groups are disproportionately affected by the COVID-19 pandemic. It is unknown whether deprivation mediates this excess ethnic risk.

**Methods:**

We used UK Biobank with linked COVID-19 outcomes occurring between 16th March 2020 and 24th August 2020. A four-way decomposition mediation analysis was used to model the extent to which the excess risk of testing positive, severe disease and mortality for COVID-19 in South Asian and black individuals, relative to white individuals, would be eliminated if levels of high material deprivation were reduced within the population.

**Results:**

We included 15 044 (53.0% women) South Asian and black and 392 786 (55.2% women) white individuals. There were 151 (1.0%) positive tests, 91 (0.6%) severe cases and 31 (0.2%) deaths due to COVID-19 in South Asian and black individuals compared with 1471 (0.4%), 895 (0.2%) and 313 (0.1%), respectively, in white individuals. Compared with white individuals, the relative risk of testing positive for COVID-19, developing severe disease and COVID-19 mortality in South Asian and black individuals were 2.73 (95% CI: 2.26, 3.19), 2.96 (2.31, 3.61) and 4.04 (2.54, 5.55), respectively. A hypothetical intervention moving the 25% most deprived in the population out of deprivation was modelled to eliminate between 40 and 50% of the excess risk of all COVID-19 outcomes in South Asian and black populations, whereas moving the 50% most deprived out of deprivation would eliminate over 80% of the excess risk of COVID-19 outcomes.

**Conclusions:**

The excess risk of COVID-19 outcomes in South Asian and black communities could be substantially reduced with population level policies targeting material deprivation.

## Introduction

Coronavirus disease-2019 (COVID-19) is an infectious disease caused by the SARS-CoV-2 virus. Mounting evidence suggests that people from minority ethnic groups in the UK (predominantly South Asian and black African or Caribbean populations) and elsewhere are disproportionately affected by COVID-19 with a higher risk of infection, hospitalization and mortality.^1–5^ The reasons for these ethnic disparities are unclear. 

Material deprivation is a universal underpinning determinant of health inequalities within and between populations.^6^^,^^7^ It has further been suggested that factors linked to material deprivation may also be important in understanding the excess risk of COVID-19 observed in minority ethnic communities.^4^ This hypothesis has not been rigorously investigated. Several studies have reported that the risk of COVID-19 outcomes in ethnic minorities is independent of potential confounders including deprivation,^8^^,^^9^ while other studies have reported the opposite finding.^10^ A recent analysis of population level data from the UK concluded that the elevated risk of COVID-19 mortality in South Asian and black populations was largely explained by demographic and socio-economic factors closely linked to deprivation,^3^ with conclusions based on the degree of attenuation with adjustment. However, these findings only provide estimates of risk when markers of deprivation are held at a fixed value; they do not quantify the degree to which the excess risk in ethnic minority groups could be eliminated by reducing material deprivation using formal mediation analysis frameworks.

We conducted a mediation analysis to model how much of the excess risk in testing positive for COVID-19, developing severe disease or COVID-19 mortality in South Asian or black individuals compared with white individuals would be eliminated if levels of material deprivation were reduced.

## Methods

We used UK Biobank, a large prospective cohort of middle-aged adults designed to support health research focused on improving the prevention, diagnosis and treatment of chronic diseases. Between March 2006 and July 2010, individuals living within 25 miles of one of the 22 study assessment centres located throughout UK, Scotland and Wales were recruited and attended data collection.^11^ Ethical approval was obtained from the North West Centre for Research Ethics Committee (Ref: 11/NW/0382); all participants provided informed consent.

### Outcomes

UK Biobank data are linked to Public Health England’s Second Generation Surveillance System for SARS-CoV-2 laboratory test data collected throughout UK.^12^ Data were available for the period 16th March 2020 to 24th August 2020 and included the outcome (positive, negative) of the test, as well as whether the specimen was collected within a hospital testing. For the purposes of this analysis, we classified a positive test result from an in-hospital setting as defining severe COVID-19 cases, as proposed through the linkage method.^12^ Furthermore, we additionally investigated COVID-19 mortality and defined a COVID-19-related death as any death with ICD-10 code U07.1 or U07.2 as the primary cause of death on the death certificate using national mortality records through NHS Digital. Death data were available up to 24 August 2020. As linked testing data are only available for participants based in UK, those from Wales and Scotland were not included in this analysis. Those who died before the first COVID-19 testing sample date (16 March 2020) were also excluded. Supplementary figure S1 shows the selection of participants.

### Exposure

Ethnicity was self-reported using a touch screen questionnaire. For this analysis, those classifying themselves as white (British, Irish, white or any other white background), South Asian (Asian or Asian British: Indian, Pakistani or Bangladeshi) or black (black or black British: Caribbean, African or any other black background) were included. South Asian and black individuals (SAB) were analysed as a single ethnic minority group. This approach was subject to a sensitivity analysis (details are reported in the statistical methods). Other minority ethnic groups, including those reporting a mixed ethnicity, were not included due to low numbers and evidence that the risk of COVID-19 outcomes is highest for SAB individuals.^2–4^

### Mediator

The mediator in this analysis was considered as material deprivation status, measured by the Townsend score,^13^ a composite of four domains: unemployment, non-car ownership, non-home ownership and household overcrowding. Within UK Biobank, data for each domain are taken from the UK Census (2001) that preceded the start of recruitment (2006) with output at the postcode area level. Each domain is given equal weighting. The resulting total score is log transformed and standardized to the UK population. A higher score represents greater deprivation. It has previously been shown that the Townsend score at the area level correlates strongly with measures of deprivation at the individual level.^14^

### Selected confounders

Age, measured at 16 March 2020, and sex were included as covariates in this analysis due to lower age and proportion of women in the SAB population within UK Biobank. Additional confounders were not considered due to the mediation model and pathway specified.

Specifically, mediation analyses assume that: (i) there is no exposure-outcome confounding; (ii) there is no mediator-outcome confounding; (iii) there is no exposure-mediator confounding and (iv) no mediator-outcome confounder is itself affected by the exposure. When ethnicity is set as the exposure, assumptions (i) and (iii) hold *a priori* as only unmeasured historical or genetic factors are true potential confounds of the construct of ethnicity. For assumption (ii), traditional confounders such as health behaviour or chronic disease are implausible confounders of deprivation and may further violate assumption (iv) as both can be argued to be influenced by ethnicity.^15^^,^^16^ Implausibility of confounders for deprivation within the model follows from a hypothesis supported by the literature that inequalities in health or health behaviours in people living with high deprivation are, in the most part, the result of high deprivation itself.^17^^,^^18^ The DAG shown in Supplementary figure S2 illustrates this concept in detail.

### Statistical analysis

Analysed outcomes were testing positive for COVID-19, developing severe (inpatient) disease or COVID-19 mortality. The population with each confirmed outcome was compared with the overall linked population without the outcome, as previously described.^19^

To test the mediating effect of deprivation in the association of ethnicity with COVID-19 outcomes, we used the four-way counterfactual approach proposed by Vanderweele,^20^ applied using regression models through the user-written *med4way* command in Stata,^21^ adjusted for age and sex. The counterfactual approach provides a framework for determining the strength of the direct and indirect pathways and their possible interactions through decomposing the excess relative risk into four components, described as the controlled direct effect (CDE), reference interaction (INT_ref_), mediated interaction (INT_med_) and the pure indirect effect (PIE). The counterfactual notation for each output is displayed in Supplementary table S1, with the underpinning mathematical formulae presented elsewhere.^20^

From a public health perspective, the INT_ref_, INT_med_ and the PIE can be summed to model the proportion of the excess risk in the exposure that would be eliminated at a fixed level of the mediator.^20^^,^^22^ For this analysis, the mediator–material deprivation, was included as a binary variable, categorised at the 75th percentile of the Townsend score (value = 0.4) defining the most deprived quarter of population. The outputs from the model can therefore be interpreted as the degree to which the excess risk in SAB, compared with white populations, would be eliminated following a hypothetical intervention where the most deprived within the UK Biobank population were moved to below the 75th percentile of the Townsend score.^20^^,^^22^ We repeated the analysis when defining deprivation status at the median value (Townsend Score = −2.2).

A sensitivity analysis was also conducted for testing positively for COVID-19 and severe disease when South Asian and black populations were considered separately; mortality analysis was not stratified due to the limited number of events. Results are reported with 95% CI unless reported otherwise.

The code for the analysis and to identify the population, main exposure, mediator, covariates and outcomes are available on GitHub (razieh93).

## Results

Supplementary figure S1 shows the flow diagram of individuals included for this analysis while table 1 reports the study characteristics. The analysis included 15 044 SAB ethnic minority and 392 786 white individuals. Subjects of SAB ethnic minorities had a median (IQR) age of 62.1 (56.3, 69.5) years and were younger than white individuals [68.8 (61.2, 74.0) years]. In total, there were 151 (1.0%) positive tests, 91 (0.6%) severe cases and 31 (0.2%) deaths due to COVID-19 cases in SAB individuals compared with 1471 (0.4%), 895 (0.2%) and 313 (0.1%), respectively, in white individuals. Deprivation scores were higher in SAB individuals, with a median (IQR) score of 1.4 (−1.2, 4.1) compared with −2.3 (−3.7, −0.2) for white individuals. The distribution of deprivation within each ethnic group is displayed in Supplementary figure S3.

**Table 1:** Cohort characteristics

	**White (*n* = 392** **786)**	**South Asian and Black (*n* = 15** **044)**	**Total (*n* = 407** **830)**
Age (years)	68.8 (61.2, 74.0)	62.1 (56.3, 69.5)	68.6 (60.9, 73.9)
Women	216** **874 (55.2%)	7978 (53.0%)	224** **852 (55.1%)
Men	175** **912 (44.8%)	7066 (47.0%)	182** **978 (44.9%)
Deprivation (Townsend score)	−2.3 (−3.7, 0.2)	1.4 (−1.2, 4.1)	−2.2 (−3.7, 0.4)
Positive cases	1471 (0.4%)	151 (1.0%)	1622 (0.4%)
Severe disease	895 (0.2%)	91 (0.6%)	986 (0.2%)
COVID-19 mortality	313 (0.1%)	31 (0.2%)	344 (0.1%)

Data as number (%) or median (IQR).

**Figure 1:**
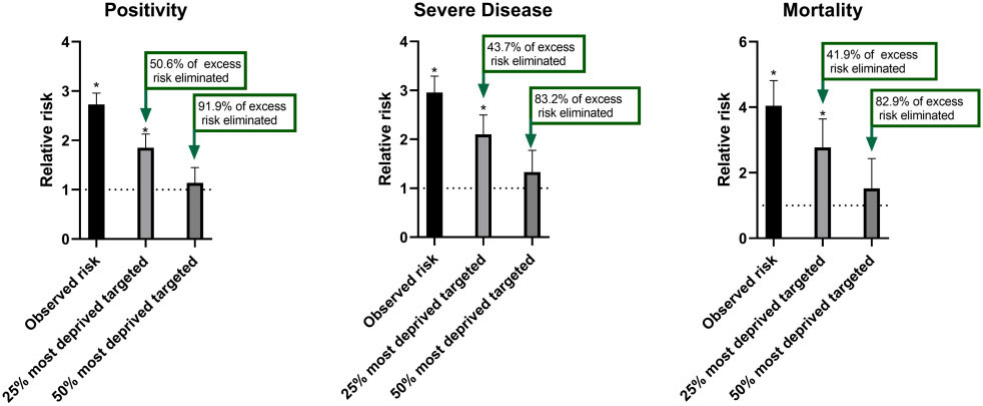
Modelling showing the relative risk of COVID-19 outcomes in black and South Asian relative to white ethnicities and the degree to which the risk is eliminated if the most deprived 25% or 50% in the population were moved out of deprivation. Data represent relative risk compared with white ethnicities. Error bars represent the standard error. Dotted line represents the reference (white ethnicities) *P < 0.05

Compared with white individuals, the age and sex-adjusted relative risk of testing positive for COVID-19, developing severe disease and COVID-19 mortality in SAB individuals were 2.73 (95% CI: 2.26, 3.19), 2.96 (2.31, 3.61) and 4.04 (2.54, 5.55), respectively. The results from the mediation analysis are displayed in Supplementary table S2. The combined PIE, INT_med_ and INT_ref_ suggest that moving the 25% most deprived in the population out of deprivation would substantially reduce the relative risk in SAB compared with white populations, eliminating between 40 and 50% of the excess risk for all COVID-19 outcomes (figure 1; data in Supplementary table S2). When modelling the impact of moving the 50% most deprived in the population out of deprivation, over 80% of the excess risk in all COVID-19 outcomes were eliminated, with SAB individuals no longer having a meaningfully higher risk (figure 1; data in Supplementary table S2).

The proportion of the excess relative risk eliminated through targeting deprivation was similar when South Asian and black populations were analysed separately (Supplementary figure S4).

## Discussion

This analysis provides novel evidence suggesting that interventions aimed at reducing material deprivation within the whole population could act to substantially reduce ethnic inequalities in the risk of COVID-19 outcomes. Specifically, a hypothetical intervention to move the 25% most deprived out of material deprivation would eliminate 40–50% of the relative excess risk for developing COVID-19 outcomes in SAB populations compared with white populations. A more extreme intervention to move the 50% most deprived out of material deprivation would eliminate over 80% of the excess risk. These findings suggest the central importance of material deprivation in driving ethnic inequalities for COVID-19 outcomes.

To our knowledge, this is the first analysis to apply a counterfactual mediation model to the role that deprivation plays in the risk of COVID-19 outcomes in SAB communities. Previously, studies have used deprivation as a covariate in logistic regression models with some concluding that the risk of COVID-19 outcomes are independent of deprivation.^8^^,^^9^ A larger population level analysis of UK data suggested the opposite,^3^ concluding that adjusting for factors linked to deprivation attenuated much of elevated risk of COVID-19 mortality in SAB individuals. These studies cannot be used to quantify the portion mediated by material deprivation or eliminated if material deprivation was reduced.

Key strengths of this study are the application of Vanderweele’s four-way decomposition model to a large contemporary population linked to COVID-19 outcomes, with the outputs designed to allow public health inferences around mediation and elimination of risk in the exposure by changing the mediator.^20^^,^^22^ There are also potential limitations that should be highlighted. Although well defined, the Townsend score assesses material deprivation across four narrow domains at the postcode area level. Material deprivation is a complex construct that is likely to be influenced at the individual level by a wide range of factors beyond those assessed. Therefore, the analysis will not capture the impact of material deprivation in its entirety. The mediation analysis is also contingent on the model assumptions, including the lack of adjustment for mediator-outcome confounders given that deprivation was postulated to be a stronger driver of health and health behaviours than the other way around. It is also acknowledged that rather being confounders, factors like chronic disease and health behaviours could potentially act as additional mediators between ethnicity and COVID-19 outcomes, and thus more complex models may be needed to examine whether a greater proportion of the excess risk in SAB individuals could be explained through additional mediation pathways compared with that explained by material deprivation alone. The observational design of this study means the findings from the mediation analysis are subject to the usual caveats around causality; results should therefore be interpreted as modelled values based on observational data rather than supporting definite direct causal inferences. The UK Biobank cohort has been noted to be healthier and more affluent than the national average, with a lower proportion of SAB individuals^23^; however, relative comparisons of associations within the cohort are still informative.^24^ Indeed, the relative risk in the ethnic minorities included in this analysis were consistent with recently reported estimates from a larger population level study,^3^ suggesting the excess relative risk examined as part of the mediation analysis is consistent with the excess risk observed in the general population.

The comparator population used in the analysis also has strengths and limitations.^17^ While cases of severe disease or COVID-19 mortality should have been picked up through the linkage system, the comparator population would nevertheless have contained those with undiagnosed mild or asymptomatic disease. In particular, during the first wave of the pandemic in UK, it is estimated that the majority of cases remained undetected,^24^ with early testing policies limited to clinical need or health care professionals. This can lead to collider bias, particularly when a positive test is used as the outcome.^19^ However, this cause of bias will be less relevant to the harder outcomes of severe (in-hospital) disease or mortality when the whole cohort without these outcomes is used as the comparator.^19^ It is also possible that some reported cases may have resulted from a false-positive, although false-positive rates in UK are estimated to be low at between 0.8% and 4.0%.^25^ The findings should therefore be interpreted as the UK Biobank population level risk of testing positive for COVID-19 within the national testing frameworks, developing severe disease or COVID-19 mortality, but not as the risk of overall infection or exposure. As this is an evolving pandemic, data should be interpreted as relating to the first wave of the pandemic in UK only. It is also acknowledged that the terms ‘black African or Caribbean’ and ‘South Asian’ cover a wide range of different cultures, languages and religions and possess fundamental differences in their physiological makeup. Consequently, our results may not apply to all black African or Caribbean and South Asian populations. However, comparisons using these ethnic groupings are still informative for understanding initial ethnic differences that can then be further investigated and stratified.

In conclusion, these results suggest reducing levels of material deprivation within the whole population could potentially play a pivotal role in reducing ethnic inequalities in COVID-19 outcomes observed South Asian and black communities. This further highlights the central role that deprivation is likely to play in driving ethnic health inequalities and the importance of policies working to reduce levels of deprivation within the whole population.

## Supplementary data

Supplementary data are available at *EURPUB* online.

## Supplementary Material

ckab041_Supplementary_Data
