# Supplementary material for: Ethnic minorities and COVID-19: examining whether excess risk is mediated through deprivation
Source: Eur J Public Health. 2021 Apr 7;31(3):630–4. doi: 10.1093/eurpub/ckab041 (PMC8083789; doi:10.1093/eurpub/ckab041)
Supplement: ckab041_Supplementary_Data [file ckab041_supplementary_data.docx]

**Supplementary Table S1. Counterfactual notations from the four-way decomposition model and their interpretation in relation to investigating deprivation as a mediator of the excess risk of COVID outcomes in South Asian and black (SAB) individuals relative to white (W) individuals.**

| **Parameter** | **Counterfactual definition*** | **Statistical definition** | **Interpretation in the context of this analysis** |
| --- | --- | --- | --- |
| Controlled direct effect (CDE) | (*Y*_sab↓_ - *Y*_w↓_) | Due to neither mediation nor interaction | Excess risk of COVID-19 outcomes in SAB relative to W individuals if both ethnic groups are modelled to live with low levels of deprivation |
| Reference interaction (INT_ref_) | (*Y*_sab↓_- *Y*_w↓_ -*Y*_sab↑_ + *Y*_w↑_) M_↑_ | Due to interaction only | Excess risk of COVID-19 outcomes acting through the additive interaction between deprivation and ethnicity that does not contribute to mediation |
| Mediated interaction (INT_med_) | (*Y*_sab↓_- *Y*_w↓_ -*Y*_sab↑_ + *Y*_w↑_) M_↑_ - M_↓_ | Due to mediation and interaction | Excess risk of COVID-19 outcomes acting through the interaction between ethnicity and deprivation when considering the effect of SAB ethnicity on deprivation. Effect contributes to mediation |
| Pure indirect effect (PIE) | (*Y*_w↓_ - *Y*_w↑_) M_↑_ - M_↓_ | Due to mediation only | Proportion of excess risk of COVID-19 outcomes mediated solely by the difference in deprivation observed between W and SAB |

*Where Y_xm_ is the value of the outcome (Y) if the exposure (X) were set to x [either SAB or W] and mediator (M) is set at level ↓ (low deprivation) or ↑ (high deprivation).

M_↑_ represents the additive interaction that operates only when deprivation is present and ethnicity is set to W.

M_↑_ - M_↓_ represents the total effect on deprivation if ethnicity is set to BAC.

The formulae are taken from VanderWeele’s *empirical analogs* [reference 18 in the main text]. The overall relative risk (RR) in SAB relative to W is equivalent to CDE + INT_ref_ + INT_med_ + PIE +1. The total proportion of the excess risk eliminated (TPE) if high derivation is eliminated is derived by summing PIE + INT_med_ + INT_med_.

**Supplementary Table S2: Outputs from the four-way decomposition model.**

|  |  |  | **Percentage (%) of excess risk acting through each decomposition if the 25% most deprived are targeted** | | | | | **Percentage (%) of excess risk acting through each decomposition if the 50% most deprived are targeted** | | | | |
| --- | --- | --- | --- | --- | --- | --- | --- | --- | --- | --- | --- | --- |
| **Outcome** | **Overall RR** | **Excess RR** | CDE | INT_ref_ | INT_med_ | PIE | **TPE** | CDE | INT_ref_ | INT_med_ | PIE | **TPE** |
| **Confirmed COVID-19** | 2.73  (2.26, 3.19) | 1.73  (1.26, 2.19) | 49.4  (22.4, 76.4) | 15.8  (5.0, 26.6) | 23.6  (7.4, 39.8) | 11.2  (7.2, 15.2) | **50.6**  **(23.6, 77.6)** | 8.1  (-26.5, 42.7) | 49.1  (28.8, 69.4) | 34.2  (20, 48.3) | 8.6  (5.6, 11.6) | **91.9**  **(57.3, 126.5)** |
| **Severe COVID-19** | 2.96  (2.31, 3.61) | 1.96  (1.31, 2.61) | 56.3  (23.1, 89.4) | 12.8  (-0.01, 26.3) | 19.1  (-0.01, 39.1) | 11.8  (6.8, 16.8) | **43.7**  **(10.6, 76.9)** | 16.8  (-26.3, 59.8) | 43.9  (18.5, 69.2) | 8.8  (5.2, 12.5) | 39.3  (21.4, 57.3) | **83.2**  **(40.1, 126.3)** |
| **COVID-19 mortality** | 4.04  (2.54, 5.55) | 3.04  (1.54, 4.55) | 58.1  (10.2, 106.0) | 13.1  (-6.2, 32.4) | 19.6  (-9.4, 48.6) | 9.2  (3.4, 15.0) | **41.9**  **(-6.0, 89.8)** | 17.1  (-39.9, 74.1) | 45.7  (12.1, 79.2) | 31.8  (8.4, 55.1) | 5.5  (1.9, 9.1) | **82.9**  **(25.9, 139.9)** |

Data shown with 95% CI. CDE = Controlled direct effect, INTref = Reference interaction, INTmed = Mediated interaction, PIE = Pure indirect effect, **TPE = total proportion eliminated**.

**Supplementary Figure S1. Flow of participants.**

Other or no ethnic coding removed
(n = 12,240)

White, South Asian or black participants with deprivation data (n = 407,830)

South Asian and black participants (n = 15,044)

Age = 62.1 (56.3, 69.5)

Women = 7978 (53%)

Men = 7066 (47%)

White participants
(n = 392,786)

Age = 68.8 (61.2, 74.0)

Women = 216, 874 (55%)

Men = 175,912 (45%)

Did not have deprivation data
(n = 469)

Scottish and Welsh participants removed (n = 56,649)

Restricted to English centres for data linkage (n = 445,855)

Historical mortality cases removed (n = 25,316)

Participants alive as of 16^th^ March 2020 (n = 420,539)

White, South Asian or black participants (n = 408,299)

Full UK Biobank cohort (n = 502,504)

Age is reported as median (IQR).

**Supplementary Figure S2: Directed acyclic graph (DAG) showing the pathways analysed and the likely impact of potential confounders not considered within the analysis.**

**
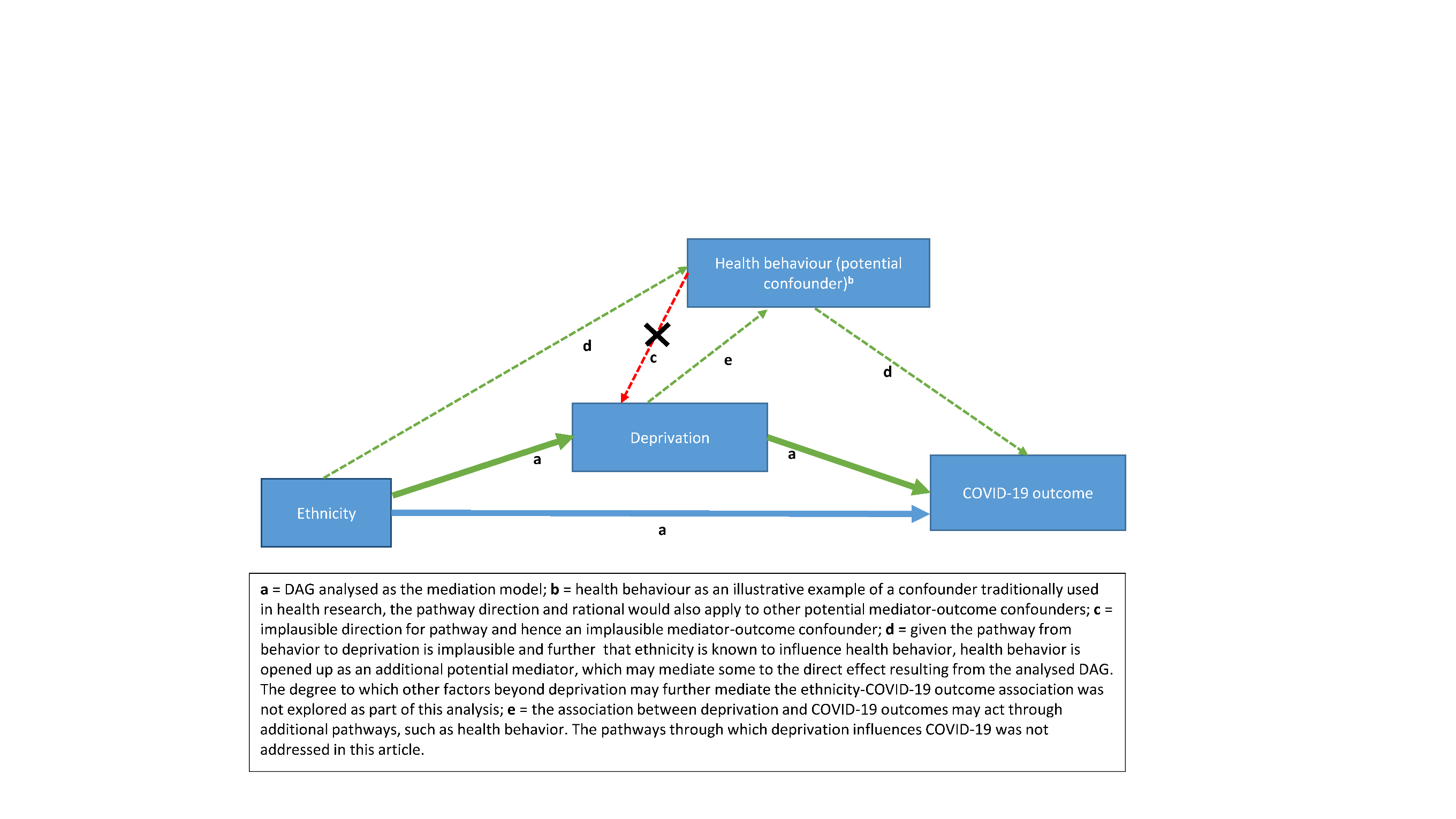
**

**Supplementary Figure S3: Distribution of deprivation within the South Asian, black and white populations.**

**
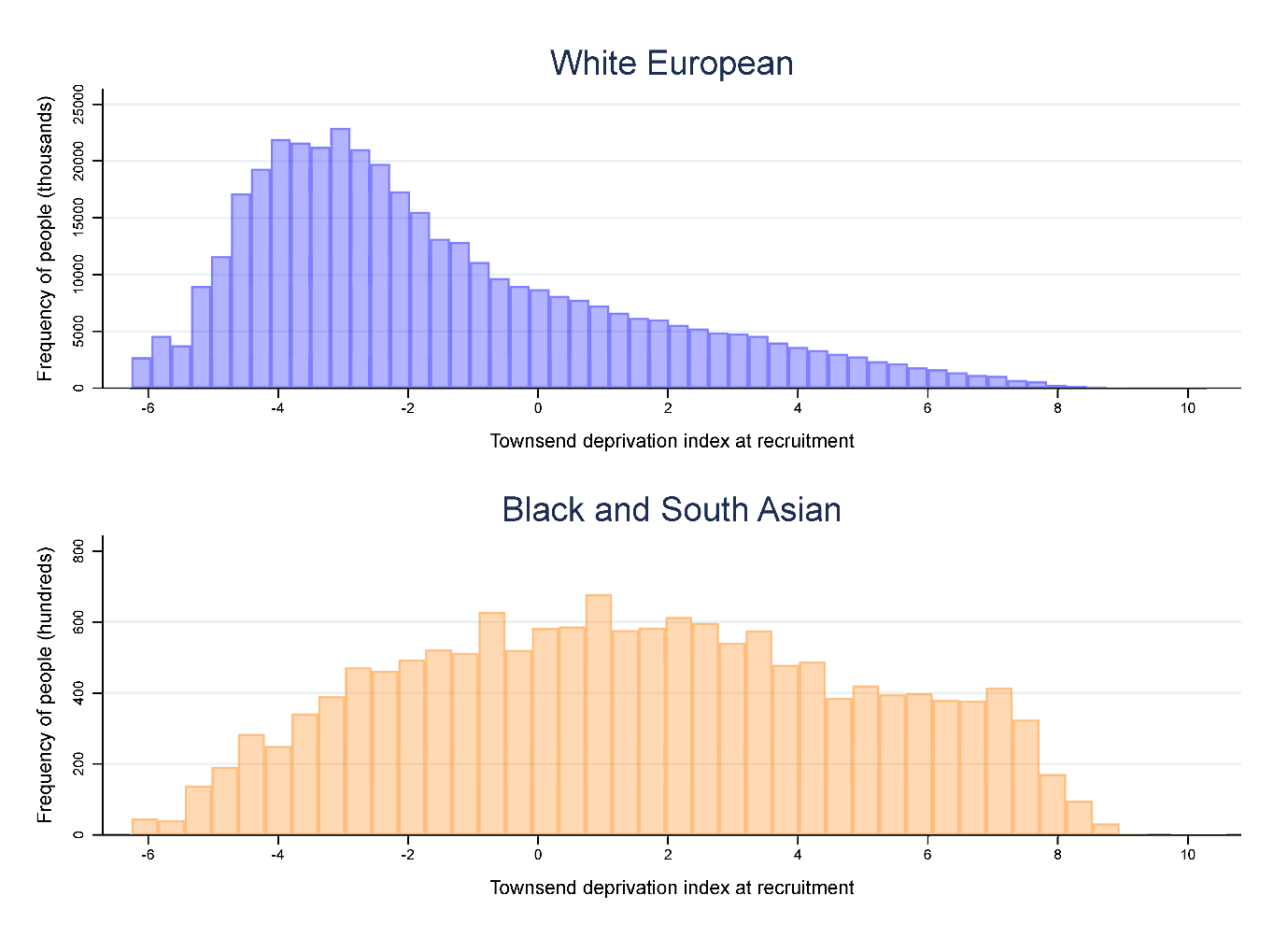
**

Higher deprivation

Lower deprivation

**Supplementary Figure S4: Results for COVID-19 positivity and severe disease when stratified by minority ethnic group.**

**
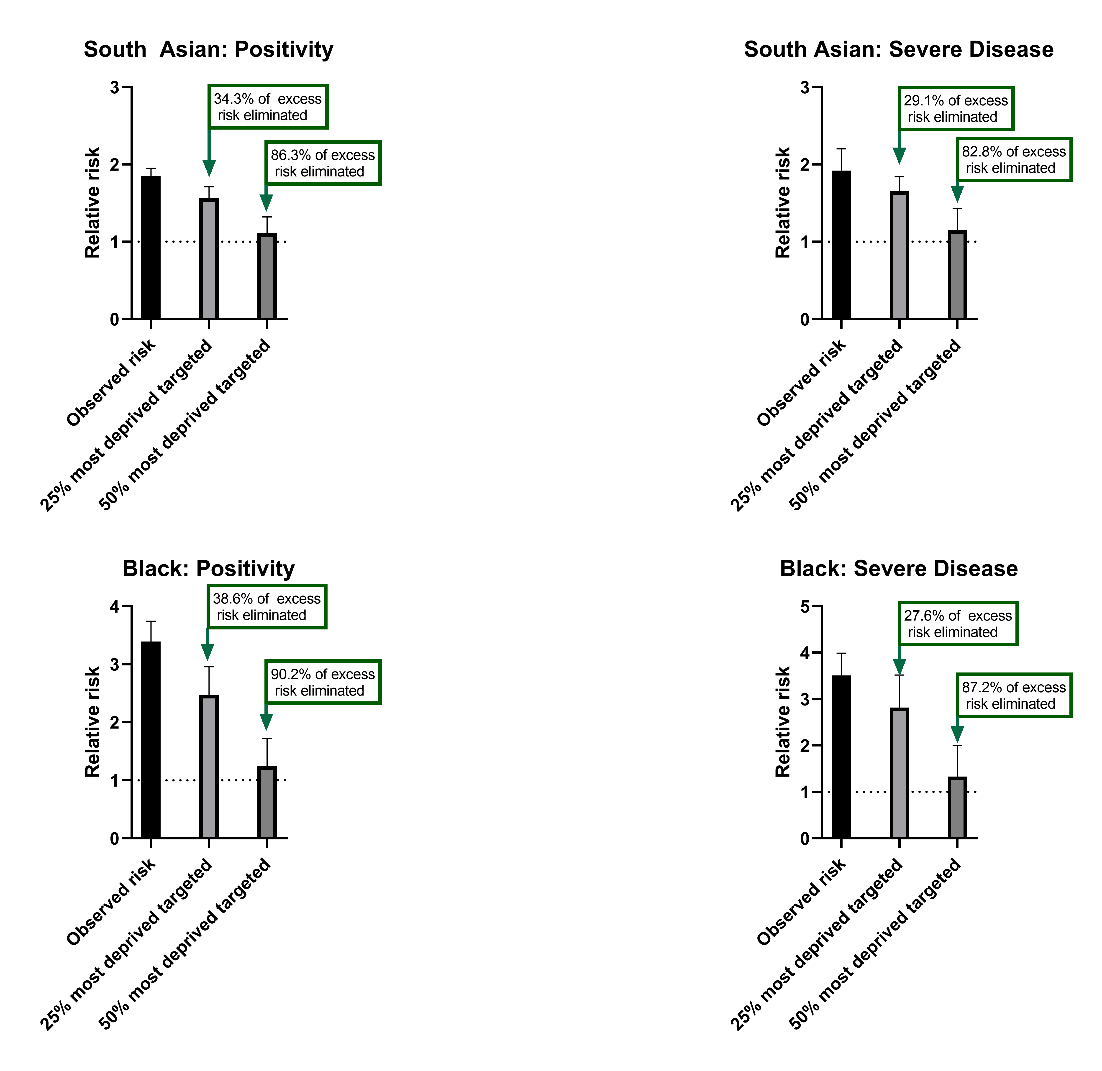
**

Data represent relative risk compared to white ethnicities. Error bars represent the standard error.
